# Evaluation of the implementation of an intervention to improve the street environment and promote walking for transport in deprived neighbourhoods

**DOI:** 10.1186/s12889-017-4637-5

**Published:** 2017-08-14

**Authors:** Emma J. Adams, Nick Cavill, Lauren B. Sherar

**Affiliations:** 1 National Centre for Sport and Exercise Medicine, School of Sport, Exercise and Health Sciences, Loughborough University, Loughborough, LE11 3TU UK; 2 Cavill Associates Ltd, 185A Moss Lane, Bramhall, Stockport, Cheshire, SK7 1BA UK

**Keywords:** Implementation, Walking, Active transport, Physical activity, Built environment, Street environment

## Abstract

**Background:**

Levels of physical activity remain low, particularly in deprived areas. Improving the street environment to promote walking for transport using a community engagement approach is a potential strategy to increase physical activity. An understanding of the implementation of this intervention approach is needed to facilitate further research, replication and scale-up. The aim of this study was to evaluate the implementation of the Fitter for Walking (FFW) intervention in deprived neighbourhoods.

**Methods:**

FFW was delivered in five regions of England between August 2008 and March 2012 and aimed to use a community engagement approach to improve the street environment to promote walking for transport. Implementation was assessed in relation to reach; dosage; implementation processes and adaptation; and factors influencing implementation. Three data sources were used: focus groups and face-to-face interviews with coordinators; implementation logs; and participation records.

**Results:**

*Reach:* 155 community groups participated in FFW engaging 30,230 local residents. *Dosage:* A wide variety of environmental improvements were implemented by local authorities (LAs) (42 projects) and by communities (46 projects). Examples of LA-led improvements included removal of encroaching vegetation, new/improved pedestrian signage, new dropped kerbs/kerb improvements and new, repaired or improved footpaths. Examples of community-led improvements included planting bulbs, shrubs or bedding plants, clean-up days and litter pick-ups. In 32 projects, no environmental improvements were implemented. Promotional and awareness-raising activities were undertaken in 81 projects. Examples included led walks, themed walks, development of maps/resources to promote improved routes and community events. *Processes and adaptation:* The need for a planning phase, a preparatory phase, and a delivery phase with a four step process were identified. Adaptability to local context was important. *Factors influencing implementation:* Five key themes were identified in relation to the barriers and facilitators of implementing FFW: local knowledge and contacts; intervention delivery; coordinator role; working with LAs and other partners; and working with communities.

**Conclusions:**

FFW is one of few reported interventions which have used a community engagement approach to change the street environment to promote walking for transport in deprived neighbourhoods. Delivering these types of interventions is complex and requires considerable resource and time. A set of recommendations and an implementation framework are proposed for future delivery of this and similar types of programme.

**Electronic supplementary material:**

The online version of this article (doi:10.1186/s12889-017-4637-5) contains supplementary material, which is available to authorized users.

## Background

The benefits of physical activity for health are well established [1, 2]. However, a high proportion of the adult population do not meet current recommendations for participation [3]. In particular, adults living in deprived areas have lower levels of physical activity and worse health compared to those living in more affluent areas [3–5]. The costs to the economy and healthcare from high levels of physical inactivity and associated non-communicable disease are substantial [6]. Therefore, identifying strategies to increase physical activity and improve health which can be effectively implemented in deprived areas is an important area of research.

Walking is known to have benefits for health [7–12]. In addition, as it is free and does not require special skills or equipment, walking has been described as near “perfect exercise” [13]. When used for transport purposes, walking can also: reduce transport costs, pollution and traffic congestion; improve road safety; and improve the wider determinants of health inequalities [1, 4]. In ecological models, the physical environment is identified as an important influence on physical activity including walking [14]. It has been noted that “people living in deprived areas may be de-motivated from walking due to neglected local environments” [15] therefore interventions which target environmental improvements in these areas are needed.

There is substantial evidence to support the relationship between walking for transport and attributes of the neighbourhood environment [16–20]. However, implementing large-scale community changes to the built environment (such as new road layouts or new bridges to improve connectivity for walking) requires a high level of investment and considerable time to change the urban landscape [21]. One potential lower cost solution is to change the neighbourhood environment at the street level, for example on key local routes within a neighbourhood, and to make smaller scale changes which can be undertaken more rapidly (for example improved lighting, improvements to crossings such as dropped kerbs, improved and maintained footpaths, traffic calming measures e.g. 20 mile per hour zones or speed bumps, or improving the aesthetics of the route [21–23]). These types of changes have shown some potential for increasing walking or overall physical activity. However, only a small number of studies have been undertaken [21, 24] and few have been reported in the scientific literature which have specifically targeted deprived areas or engaged communities in identifying the environmental changes needed.

Community engagement can be defined as the “direct or indirect process of involving communities in decision making and/or in the planning, design, governance, and delivery of services using methods of consultation, collaboration, and/or community control” [25]. This approach can potentially help to improve health and well-being and reduce health inequalities [26]. Engaging local communities in identifying local environmental barriers to walking and potential solutions is one possible approach for improving the street environment ensuring local needs and issues are addressed, and relevant and rapid improvements made. The authors are aware of only one reported study undertaken in the US which used a community engagement approach to instigate and undertake street scale environmental changes [27]. This study was effective in increasing walking but no evaluation of implementation was undertaken, making it difficult to identify effective implementation strategies or replicate the intervention. Although some community-wide interventions have included the use of a community engagement approach to make environmental changes as part of a wider programme of activities [28], there has been no separate reporting of the implementation and effectiveness of these specific interventions. Therefore further studies are needed to assess the potential of this approach for improving the street environment and promoting walking, both in terms of implementing this type of strategy and determining effectiveness for increasing walking levels.

Implementation research aims to assess “the types and quantities of policies and interventions delivered, the beneficiaries of those policies and interventions, the resources used to deliver the policies and interventions, the practical problems encountered, and the ways in which such problems were resolved” [29]*.* It is particularly focussed on research related to the implementation of interventions in real world settings [30]. Evaluating and understanding the processes involved in implementing interventions in real world settings is important in order that effective implementation strategies can be identified enabling interventions to be improved, replicated, scaled-up and embedded into local systems. The field of implementation research is relatively young, particularly in relation to the study of physical activity and public health interventions, and to date this has been a largely under-studied area. The need to undertake and publish evaluations of real world interventions is a priority for physical activity research [31].

To the authors’ knowledge, only five studies have considered the factors influencing the implementation of community-based walking interventions [23, 32]. Most of these studies did not undertake a comprehensive evaluation of implementation, and none specifically assessed interventions which aimed to implement environmental improvements using a community engagement approach. The aim of this study was to evaluate the implementation of the ‘Fitter for Walking’ (FFW) intervention in order to inform future research, practice and policy with regard to using a community engagement approach to change the street environment to promote walking for transport in deprived neighbourhoods. The objectives of the evaluation were to assess: 1) recruitment and participation in the intervention; 2) what was delivered as part of the intervention; 3) how the intervention was delivered; and 4) the barriers and facilitators for implementation.

## Methods

### 'Fitter for Walking' intervention 

**Fig. 1:**
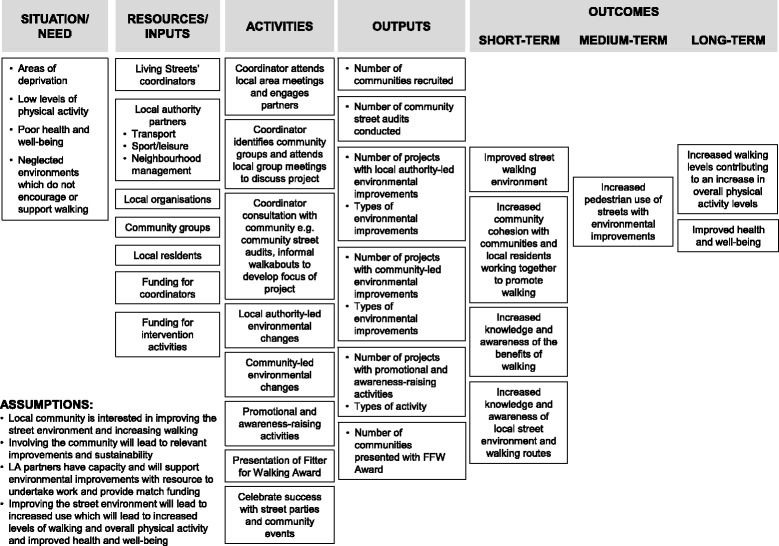
Logic Model for Fitter for Walking

FFW was a practice-led, community-based intervention which aimed to: improve the local neighbourhood walking environment; increase the number of people walking on a specific route targeted for environmental improvements; and encourage communities and local residents to work together to promote walking. A logic model is provided in Fig. 1 to present what was planned for the intervention and to outline how short and long-term outcomes link to intervention activities, resources and the assumptions made. FFW was managed, developed and delivered by a third sector organisation based in the UK (Living Streets) between August 2008 and March 2012. Five full-time FFW coordinators were employed by Living Streets, one in each region, to engage local communities, facilitate community relationships with the LA partner, develop local partnerships, identify additional local funding and support the environmental improvements and awareness-raising activities which were undertaken. The coordinators met every 3 months to discuss progress, and share experiences and ideas. One of the coordinators also acted as the Project Manager with overall responsibility for the FFW intervention. Twelve LA partners were recruited from five regions of England to take part (Table 1). The LA areas recruited had low levels of reported physical activity based on survey results from Active People Survey 1 (2005–2006) [33] and were based in areas of high deprivation (see Additional file 1). FFW was based within the transport department in eleven of the twelve LAs and in the sports/leisure department in the remaining LA. The role of the LA was to work with coordinators to identify and select suitable neighbourhoods/community groups in which to deliver the intervention, provide funding and undertake environmental improvements (identified by the communities) to promote walking. All twelve LAs remained involved in FFW until the overall intervention funding came to an end in March 2012.

**Table 1:** Fitter for Walking projects by region and local authority

Region	Local Authority	Project status^a^									
		Number of projects									
		Primary groups^b^	Secondary groups^c^	Total	Number of groups registered^d^	Planned^e^	Completed^f^	In progress/ongoing^g^	On hold^h^	Declined to participate^i^	Withdrew post-registration^j^
London	Barking & Dagenham	4	8	12	8	0	7	3	1	0	1
	Redbridge	8	3	11	8	0	1	6	4	0	0
	TOTAL	12	11	23	16						
North East England	Gateshead	5	7	12	12	0	8	3	1	0	0
	Newcastle	4	2	6	6	0	5	1	0	0	0
	Sunderland	5	0	5	4	1	2	2	0	0	0
	TOTAL	14	9	23	22						
North West England	Blackburn with Darwen	11	14	25	8	5	5	9	6	0	0
	Bolton	10	6	16	6	4	2	4	5	1	0
	TOTAL	21	20	41	14						
West Midlands	Dudley	7	1	8	8	0	0	5	1	0	2
	Sandwell	9	4	13	13	0	3	5	0	0	5
	Wolverhampton	6	5	11	8	1	3	3	2	0	2
	TOTAL	22	10	32	29						
Yorkshire	Doncaster	19	1	20	15	3	8	2	0	1	6
	Rotherham	15	1	16	14	1	3	5	1	1	5
	TOTAL	34	2	36	29						
TOTAL		103 (66.5%)	52 (33.5%)	155	110	15 (9.7%)	47 (30.3%)	48 (31.0%)	21 (13.5%)	3 (1.9%)	21 (13.5%)

^a^Information provided on final implementation log (September 2011)

^b^Primary groups were the first and main group to be signed up in a community who led the initial activities and environmental changes on the specified route or area

^c^Secondary groups got involved due to their links with the primary group and then became a project in their own right or got involved at a later stage in promoting the use of the improved routes or delivering promotional and awareness-raising activities

^d^Completed an application form to be part of FFW and officially registered with the intervention

^e^Planned at the time of the final implementation log

^f^Completed and officially closed at the time when the final implementation log was provided

^g^Ongoing/had not been officially completed at the time of the final completion of the implementation log

^h^On hold at the time when the final implementation log was provided

^i^Groups showed initial interest in FFW but later decided it did not fit with their interests or plans

^j^Groups withdrawn by the coordinator or withdrew themselves after registering

The intervention aimed to recruit 228 community groups with the intention of working with each group for 6–12 months. Registration to FFW was at the community group level, rather than at the individual level. FFW did not aim to recruit a specific target population but all members of the community (defined as those who lived in the area where the registered community group operated) of all ages were eligible to participate in intervention activities. At the start of the FFW intervention there was no prescribed approach for how it should be delivered. This was left flexible for the coordinators to establish how it would work in their local area. There was a broad plan for: 1) coordinators to work with LA partners, recruit community groups, consult with groups to explore barriers to walking on a specific route or local area and report the findings including recommendations for environmental improvements to the LA; 2) the LAs to fund some or all of the environmental improvements and arrange for them to be undertaken; and 3) the community to work together to also make environmental improvements and promote walking. The consultation was to be undertaken using a Community Street Audit (CSA) [34]. This involved working with local community groups and other local stakeholders, who might include local residents, businesses traders, councillors and police community support officers. The coordinator, community group(s) and stakeholders assessed a local route on foot and identified potential barriers to walking in order to suggest improvements which would create a safe, attractive and enjoyable environment. There was also a plan to develop an award for communities to recognise success and promote sustainability but the format this would take had not been decided.

An assessment of the impact of FFW on the number of people walking on a specific route has been reported elsewhere [35]. In brief, one community project was selected from each of the five regions where FFW was being implemented. Intervention activities in each of these community projects included LA-led environmental improvements, community-led improvements and promotional and awareness-raising activities. Route user counts were conducted at baseline, after 12 months and after 14–20 months. After 12 months, a decrease in pedestrian route use overall and in four community projects was observed, however after 14–20 months, there was an increase in pedestrian route use overall and in all community projects compared to baseline.

### Evaluation of implementation

The purpose of this study was to evaluate the implementation of the FFW intervention. The study draws upon the underlying principles for implementation research, which is “the scientific study of the processes used in the implementation of initiatives as well as the contextual factors that affect these processes” [36]. In addition to implementation processes and the factors affecting implementation, this study addresses a number of constructs which have been identified as being key aspects of implementation including: reach (rate of involvement); dosage (how much of the programme has been delivered); and adaptation (changes made in the intervention during implementation) [37]. The evaluation was conducted using three different data sources: 1) interviews and focus groups with the FFW coordinators; 2) an implementation log, which was used to collect data relating to delivery for each community project; and 3) participation records. Data were collected between December 2008 and October 2011. A summary of which data sources were used to assess each of the study objectives and the implementation constructs identified above is provided in Table 2.

**Table 2:** Data sources and constructs assessed

Data source	Indicators assessed/collected	Objectives addressed	Implementation constructs assessed
Interviews/ focus groups with coordinators	• Leadership	1	Recruitment (Reach)
	• Project implementation including: context, recruitment and engagement of communities, working with local authorities and other partners, delivery of different intervention activities	3	Implementation processes (Adaptation)
	• Barriers and facilitators for implementation	4	Factors affecting implementation
	• Sustainability		
Implementation log	• Name of the registered group	1	Recruitment (Reach)
	• Key dates:		
	Date of registration, date of community street audit, date FFW award presented, end date of project		
	• Community characteristics: Target community, estimated size of the community on which the project might have an impact (number of individuals or households)		
	• Location/route characteristics:		
	The main route/area of interest and any local key destinations or trip generators		
	• Project information:		
	How the group was identified/recruited, the priorities of the group for the project		
	• Barriers to walking	2	What was delivered (Dosage)
	• Environmental improvements and activities delivered		
	• Key stakeholders and partners involved in the project		
	• Additional funding identified for project activities		
	• Challenges specific to each project		
Attendance records	• Number of people attending events, community meetings and led activities	1	Participation (Reach)
Pledge cards	• Number of pledge cards distributed/completed	1	Participation (Reach)
Route user counts	• Number of people walking on specified routes in five FFW projects	1	Participation (Reach)

#### Interviews and focus groups with FFW coordinators

Interviews and focus groups were used to assess recruitment (reach), implementation processes including adaptation, and factors influencing implementation, Coordinators took part in three focus groups which were held in December 2008, November 2009, and October 2011, and an individual face-to-face interview in October/November 2010 and October 2011 (11 interviews were completed in total) to enable implementation across the whole intervention period and within each region to be explored on an ongoing basis. Interviews and focus groups lasted 45 min to 1 h and were digitally recorded. A semi-structured guide was used to initiate and direct the discussions. Key themes discussed are outlined in Table 2.

#### Implementation log

The implementation log was used to assess recruitment (reach) and what was delivered (dosage). Data relating to intervention implementation for each community project were collected by each coordinator on a bespoke Microsoft Excel spreadsheet. This spreadsheet was developed and tested in consultation with the coordinators to ensure it was feasible to use and provided useful information for the research team and the intervention management/delivery team. The spreadsheet was completed, updated and sent to the research team by the coordinators on a monthly basis from November 2009 until September 2011. The spreadsheet was used to record details of community groups as they were engaged and to track progress as projects developed. Key indicators assessed are outlined in Table 2.

#### Participation records

Participation records were used to assess individual participation in the programme (reach). Participation was assessed at two levels: 1) individuals who engaged with the programme, defined as someone who participated in at least one programme activity and thus had increased awareness of opportunities for walking; and 2) individuals who were encouraged to walk more as part of the programme, (e.g. took part in a led walking activity, completed a pledge card or walked on the improved route). These were assessed using three different approaches: 1) records completed by the coordinators of the numbers of participants attending each intervention activity including community meetings and led activities as part of the routine monitoring of the intervention; 2) distribution of pledge cards (used to encourage community members to set goals for walking); and 3) through the use of follow-up pedestrian route user counts which were conducted in five community projects at 12 months and 14–20 months after baseline (full methodology reported elsewhere [35]).

### Analyses

All interviews and focus groups were transcribed verbatim by an independent administrator. Transcripts were read thoroughly to fully understand coordinators’ perspectives and coded in NVIVO Version 10 to group findings from each of the focus groups/interviews into themes related to those in the interview guide. Key points were extracted and information presented in relation to reach (recruitment), implementation processes and adaptation. Inductive analysis was used to identify and organise themes relating to the factors influencing implementation (barriers and facilitators) from the raw data. Findings are supported with illustrative quotes, the source of which is identified using “FG” (focus group) or “Interview” and the year in which the FG or interview was conducted (e.g. FG, 2009). Data in the implementation log included quantitative and qualitative data. Quantitative data were categorical (e.g. “*Please indicate the status of the project*?” Response selected from “Planned”, “Completed”, “In progress or ongoing”, “On hold”, “Declined to participate” or “Withdrew post-registration”). Frequencies and percentages were computed for these data. Qualitative data were open-ended questions (e.g. *“How was the group identified/recruited?”* or *“Please describe the intervention activities which took place?”*). Responses to open-ended questions were read thoroughly and a content analysis conducted to code responses and compute frequencies and percentages. These data were used to assess reach (recruitment) and dosage. Data recorded on attendance records were summed to provide the total number of people engaged in the intervention; data for the number of people attending led walks, the number of pledge cards completed and the number of route users at follow-up were summed to provide an estimate of the number of people who were encouraged to walk more. Descriptive analysis was used for these findings to report reach (participation).

## Results

Findings from the data gathered for this evaluation are presented below in relation to implementation of the FFW intervention from the coordinators’ perspectives and from records kept with regard to intervention delivery and participation.

### Reach (recruitment and participation)

Overall, the coordinators worked with 155 community groups across the five regions (Table 1). The community groups included tenants’ and residents’ associations, churches, local interest groups (e.g. allotment associations, wheelchair users groups or ‘Friends of’ groups), specific ethnic groups, community centres and schools. Data on the methods used to recruit or engage community groups for FFW were provided for 140 projects in the implementation log. The most frequently reported recruitment methods are outlined in Table 3.

**Table 3:** Most frequently reported recruitment methods and activities

	Number of projects	Percent
Recruitment methods		
Approached by a local community representative^a^	71	50.7
At a local community event	19	13.6
At a local or regional meeting (e.g. local area/community forums)	17	12.1
Coordinator approached centre, group or individual	8	5.7
Through an existing FFW project or word of mouth	8	5.7
Coordinator attended local community group meeting	7	5.0
Activities		
Local Authority-led environmental improvements		
Removal of encroaching vegetation	12	10.3
New or improved pedestrian signage	9	7.8
New dropped kerbs or kerb improvements	8	6.9
New, repaired or improved footpaths	8	6.9
Resurfacing of footpaths	8	6.9
General safety improvements (e.g. new fencing around pond)	6	5.2
Extra bollards to control traffic flow and parking	6	5.2
Installation of benches/seating	6	5.2
New or improved street lighting	5	4.3
Removal, repair or replacement of street furniture (e.g. railings); and installation of maps or noticeboards for maps	5	4.3
Community-led environmental improvements		
Planting bulbs, shrubs or bedding plants	33	28.4
Clean-up days	12	10.3
Litter pick-ups	8	6.9
Clearance of land or encroaching vegetation	7	6.0
Promotional and awareness-raising activities		
Led walks	60	51.7
Themed walks (e.g. a history walk or nature walk)	18	15.5
Development of maps or resources to promote the improved route/area	22	19.0
Community events, fun days, celebration events and street parties^b^	19	16.4
School talks or assemblies	19	16.4
Pledge cards^c^	17	14.7
Walking challenges linked to walk to school month or week	12	10.3

^a^e.g. a neighbourhood manager, community engagement officers, local councillors or individuals from other community-based services such as head teachers, school governors, centre managers, or local residents

^b^Many included a walking element such as art activities for children linked to traffic safety, or a led or themed walk

^c^Individuals pledged to undertake a specific goal in relation to walking and wrote it down on a card

Three groups declined to participate after an initial meeting with the coordinator (due to lack of interest) and 21 groups were withdrawn by the coordinator or withdrew themselves after registration (Table 1). The reasons given for withdrawal (provided either on the implementation log or through coordinator interviews) included: the coordinator could not contact the group; FFW was being ‘used’ to address another agenda the group was interested in; the proposed project did not meet the remit of FFW; there were staff shortages to deliver activities; the LA was unable to act on the audit recommendations due to change in the economic climate; or the coordinator or local group were unable to source local funding for the activities proposed. At the end of the data collection period, 47 projects had been completed, 48 were still in progress, 21 were on hold and 15 were in the planning stage (Table 1).

Participation was assessed using attendance records, recording the distribution of pledge cards and through follow-up route user counts. A total of 30,230 local residents engaged with the FFW programme (participated in at least one activity and thus had increased awareness of opportunities for walking) either through attending events or taking part in intervention activities. In addition, 13,845 people were considered to have been encouraged to walk more as a result of attending walks delivered as part of the intervention, using pledge cards, or walking on the improved route. It was not possible to assess representativeness of participants due to the nature of the data collected.

### Dosage (what was delivered)

Community Street Audits were conducted in 50 (52%) primary projects and 9 (18%) secondary projects (secondary groups got involved due to their links with the primary group and then became a project in their own right, or got involved at a later stage in promoting the use of the improved routes or delivering promotional and awareness-raising activities). The activities delivered fell within three categories: 1) LA-led environmental changes; 2) community-led environmental changes; and 3) promotional and awareness-raising activities. Across the projects which were either completed, in progress or on hold by September 2011 (*n* = 116), 42 (36.2%) included type 1 activities, 46 (39.7%) included type 2 activities and 81 (69.8%) included type 3 activities. Of the 116 projects, 23 projects (19.8%) included activities from all three categories; 31 projects (26.7%) included activities from two categories (category 1 and category 2 (*n* = 4); category 1 and category 3 (*n* = 9); and category 2 and category 3 (*n* = 18); and 42 projects (36.2%) included activities from just one category (32 of which undertook category 3 activities only). At the time of final data collection, 20 projects (17.2%) had not yet implemented any environmental improvements or promotional and awareness-raising activities. Details of the types and numbers of activities delivered in each category were reported on the implementation log and are provided in Additional file 2. The most frequently reported activities are outlined in Table 3.

A FFW Award was developed mid-way through the intervention requiring four criteria to be met: environmental improvements made; more people perceived to be walking; community working together to improve the environment/promote walking; and demonstrated commitment to sustain improvements. A number of communities were presented with the Award (*n* = 30) recognising the achievements they made towards improving the local environment to promote walking as part of FFW.

### Implementation processes and adaptation (how the intervention was delivered)

There was no prescribed approach for the implementation of FFW at the start of the intervention therefore the implementation processes and adaptations were identified as delivery progressed. From ongoing discussions with coordinators, it became clear that there were a number of phases and processes involved in the implementation of FFW. These included a preparatory phase before commencing intervention activities and a four step delivery phase. The four step process which evolved during the intervention provided a coherent structure whilst still allowing flexibility and adaptability at the local level *“because every place you work in, it’s different, every community or neighbourhood is very different so there can’t be any prescriptive ‘this is what you do, this is what you need’” (FG, 2008)*. The structure provided a framework and principles for implementation which were followed by all coordinators supporting consistency in the delivery of the intervention:

*“I think there has been a thing about the project becoming more and more structured as we’ve gone through… so it’s kind of progressively, we’ve got a much more structured way of dealing with it, probably much more consistently across the whole country”. (FG, 2011)*

#### Preparatory phase

Coordinators reported an initial preparatory phase of approximately 6 months was needed to: 1) undertake research and develop knowledge of the local area to understand the local context and identify what was going on, including any issues that might overlap with what FFW was trying to achieve; 2) publicise and increase awareness of the FFW intervention (and walking) through attending meetings and community events, and visiting local services and amenities e.g. libraries, community centres; 3) identify contacts in the LA transport and other departments (e.g. community engagement) and establish working processes; 4) establish partnerships with other local organisations and networks (such as Neighbourhood Management teams, local police, local Councillors, Healthy Community Partnerships, Primary Care Trusts, local housing associations, local land owners (e.g. Network Rail), voluntary organisations (e.g. Groundwork, Wildlife Trust) and support services (e.g. Sure Start), and start to develop relationships to support the delivery of the intervention; and 5) start to identify potential community groups for the intervention in discussion with the LA and other partners. These activities were undertaken concurrently.

#### Delivery phase

Following the initial six month preparatory phase, coordinators commenced working with local community groups. The four step delivery phase included: 1) engage community groups; 2) develop local project; 3) deliver project activities and 4) recognise and reward success. An outline of the processes involved in each step is provided below.

##### Step 1: Engage community groups

The first step was to recruit and engage community groups in the FFW intervention. A wide variety of strategies were used by the coordinators to do this which included both reactive approaches (e.g. community groups or local organisations approached the coordinator such as a referral through a partner or via attendance at a local event or resident/community meeting), and proactive approaches (e.g. coordinators identified and approached groups by dropping in at local community centres, schools, churches or the local library). The coordinators thought both approaches were effective but in most areas a reactive approach was more commonly adopted:

*“I’ve been reactive rather than proactive and I think either way is valid but I’ve not had to go out and find somewhere or this could really do with doing…maybe I can put a community group in and around this project to get this project off the ground. It seems to be the communities that have come to me and said ‘I’ve got this specific issue in this area, can you come and look at this with me? Will you come and have a look at this path or this route?’ So I’m reacting to that rather than having to go out and seek projects and try and build something around that.” (FG, 2008)*

Coordinators indicated that cold calling or sending letters to community groups inviting them to take part in FFW generally did not elicit any response. Once engaged, the coordinator typically attended a local meeting of the community group, gave a presentation about the benefits of walking and the FFW intervention and where appropriate encouraged the group to apply and register to participate.

##### Step 2: Develop local project

The second step in the process was to consult with the local community group to select a route or area for their project to focus on, identifying key local destinations or ‘trip generators’ and the barriers to walking along with potential solutions using a CSA. At the start of the intervention, coordinators anticipated that CSAs would be undertaken with all community groups. In reality, this was not possible due to coordinator capacity to undertake CSAs and LA capacity to action and fund recommendations made. Coordinators reported that the CSAs were a useful tool for engaging communities in their local neighbourhood environment and the FFW intervention, and helped them to identify barriers to walking in their local areas and potential solutions. Once completed, a CSA report was sent to the LA for consideration for funding and implementation. In non-CSA projects, barriers to walking were identified through meetings with the registered group and local community members, or more informal walks around the local area. In these projects, plans were made for community-led improvements to the environment only. The importance of consulting with local communities and facilitating activities to be community-led was highlighted by the coordinators to ensure appropriate issues were addressed:

*“It was quite a good learning curve if you like, not to pre-empt too much what the issue is in that area, it’s a large shopping area and the walking access wasn’t great and it needed some regeneration. But the residents group weren’t interested in that at all, it was the local path that they were bothered about.” (FG, 2008)*

Coordinators also provided community groups with a FFW manual which was developed in the first year of the intervention by Living Streets and the FFW coordinators. This aimed to build skills and knowledge in community members and promote community ownership of project activities to support sustainability beyond the duration of the coordinators’ involvement. The manual contained ideas and resources for improving the walking environment and increasing awareness of walking which the community could use to undertake these activities themselves.

##### Step 3: Deliver project activities

During the first year of the intervention it became clear to the coordinators that there would be different types of local community projects depending on the needs, interests and skills of the group signing up to the intervention, the types of environmental barriers to walking and solutions identified, the capacity of the coordinator to undertake a full consultation whilst working with multiple different groups, the capacity of the LA to make environmental changes in all projects and the timescales and funding this required. Each local project therefore differed in terms of the types of environmental changes which were made. Coordinators reported that promotional and awareness-raising activities were important for: raising awareness of walking and its health benefits; increasing awareness of routes and facilities (including green space) in the local neighbourhood that many residents were not previously aware of; maintaining momentum and community involvement while waiting for the LA to make environmental improvements; promoting the environmental improvements that had been made; and engaging wider members of the community to extend the reach of the intervention (and meet the targets set by the funding agency).

##### Step 4: Recognise and reward success

At the time of the first focus group in 2008 no clear exit strategy from community projects had been planned to enable coordinators to end their involvement. It was anticipated that an award would be developed and once presented would act as a closure to the project. Coordinators reported that once the FFW Award was established (in the second year of the intervention), it played an important role in setting objectives at the start of each community project and in focussing the activities which were undertaken by each group due to the need to meet the specific criteria.

*“Now when we’re setting out what we want to achieve at the start, it’s not too vague, it’s not too ambiguous, it’s with certain criteria to hit, I think that makes it a lot easier and that’s something we’ve only developed again over the last sort of six to eight months, the criteria and the manual and things like that. That all helps, I think that will make it easier to start and finish projects.” (Interview, 2010)*

### Factors influencing implementation (barriers and facilitators)

Through interviews and focus groups with coordinators five key themes were identified in relation to the barriers and facilitators of implementing the FFW intervention. These included: local knowledge and contacts; intervention delivery; coordinator role; working with LAs and other partners; and working with communities. Further details of the barriers and facilitators are provided in Table 4 and key issues are discussed below.

**Table 4:** Barriers and facilitators for intervention implementation

Theme	Barriers/challenges	Facilitators
Local knowledge and contacts	• Lack of knowledge of the local area where coordinators were working in areas unfamiliar to them • Lack of established contacts and partners where coordinators were working in new areas	• Coordinator living in the local area, being knowledgeable about the local area, understanding local issues and agendas, having an established network of contacts
Intervention delivery	• Uncertainty at the start about how the project would be delivered • Lack of publicity materials and resources at the start of the intervention • Sending letters to community groups to invite them to participate in the intervention did not result in groups signing up • Recruiting hard to reach individuals/wider members of the community • Having one negative person in a group can have a substantial impact of the rest of the group • Limited funding and budgets cuts during the intervention • Timescales for engaging and working with communities and for making LA-led environmental improvements were much longer than expected • Maintaining momentum and community involvement whilst awaiting LA improvements • Gaining permission and approvals for intervention activities slowed progress • Difficulty in closing projects, particularly where the coordinator had become well embedded into the group • Coordinators were unable to work with the target number of groups due to the other barriers such as timescales and coordinator capacity • Recruiting the target number of individuals	• Flexibility to deliver the intervention to suit local needs and allow for local differences in context and operating processes • Development of a more structured intervention delivery pathway as FFW progressed • Developing contacts and linking in to existing local networks and organisations • Linking to existing projects e.g. urban regeneration schemes which helped provide additional local funding for FFW • Allowing the activities to be community-led • Use of resources e.g. the Community Street Audit helped with community engagement and provided credibility with LAs; the FFW manual enabled groups to develop and deliver their own activities providing a level of sustainability; the pledge cards were also a useful engagement tool; having case study examples from other FFW projects to help ‘sell’ the intervention to new groups • Distributing the audit report not only to the main contact in the LA, but also to other LA departments and community organisations helped to support implementation of the recommendations and lever funding • Coordinators had a budget of their own to spend on intervention activities which helped engage the LAs, other partners and communities • Ensuring the community group knew they were expected to take ownership of the project and not expect the coordinator to do all the work • Increasing reach of the intervention by working with secondary groups e.g. schools • The FFW award criteria played a role in providing focus for a community group; the award was important for communities in recognising their achievements
Coordinator role	• Capacity of coordinator to work across multiple areas with multiple groups simultaneously and time management • Level of work involved with each group, even those who were proactive and confident • Managing perceptions of communities regarding the coordinator role (some thought they were purely walk leaders, others expected the coordinator to do all the work instead of the community group taking ownership) • Coordinator changes in two regions may have affected established relationships with local groups and partners	• ‘Getting known’ and having a presence/visibility in the community • Being proactive in working with LA to follow up on status of audit report and actions on recommendations • Maintaining contact and providing regular communication to community groups on the status of their proposed environmental improvements
Working with local authorities and other partners	• Two LAs dropped out before FFW started due to being unable to commit resources and funding for the intervention • LA lack of understanding of the FFW intervention and their role • Some LAs wished to direct where the coordinator worked, or wanted the coordinator to work across the whole LA area which wasn’t feasible with coordinator capacity or where the intervention was most needed • All LAs functioned differently, coordinators had to understand this and work differently with each one • It took time for the coordinator to be accepted in the LA and have a full understanding of how the LA operated • LA processes and priorities did not always fit with FFW • The level of LA support varied; the key contact in the LA did not always provide sufficient support, or did not have the right contacts to support intervention activities • Changes of staff in the LAs affected intervention delivery • The level of support from councillors varied across areas • In some areas, there was resistance and negativity from local councillors regarding availability of funding and raising community expectations • Communication between LA departments was sometimes poor • Bureaucracy and paperwork was a hindrance to making quick progress with intervention activities • It became apparent that many different LA departments might have a role in improving the environment to promote walking, not just the transport department, however other departments in the LAs were not always aware of or engaged in the intervention • The timescales to get approval for work, planning decisions and environmental improvements made were much longer than expected • The transport departments sometimes had limited knowledge of what was happening on the ground in communities. Other departments became important for this e.g. neighbourhood management • In some areas LA neighbourhood management teams were disbanded during the intervention which affected delivery of FFW • Where there was no neighbourhood management structure, working with communities was much more challenging • Tracking the progress of audit reports once they had been submitted to the LAs was difficult and time consuming • In some LAs, provision of funding slowed down once the match fund target had been reached • Funding reduced during the course of the intervention due to budget cuts • Working with multiple partners, with different agendas and different ways of working was challenging	• LA being willing to provide match funding and resources to support FFW • Having the right contact in the LA facilitated intervention implementation and timescales for action • Knowing who to contact to ensure action takes place • In some areas, having a more senior member of staff helped with implementation, in others more junior members of staff were important in facilitating progress • LAs provided capacity to undertake work e.g. use of community pay back teams to clean graffiti from a bridge, or engaging the local navy to assist with a clean-up day • Having a neighbourhood management structure in place enabled coordinators to quickly access information about the local area, community groups and issues • Developing partnerships with local organisations provided additional support for FFW, increased publicity, built capacity, helped to identify community groups, ensured there was no duplication of activities and promoted long-term sustainability
Working with communities	• Some FFW projects were based in urban regeneration areas and community experiences of this (changes to timescales, promises not kept, communities felt they were not listened to, changes made the community did not want) led to some resistance to FFW • Identifying a focus for the local project, sometimes the community group did not have a clear environmental issue they wished to address and were only interested in walking • Maintaining focus on changing the environment and promoting walking for transport; some groups only wanted led walks or focussed more on improving green space and recreational walking • Some coordinators felt they were ‘used’ to set up walking groups or to address agendas which were not the focus of FFW • Managing expectations; it was not always possible to make all the environmental improvements which were requested and slow timescales • Some community groups required a lot of support impacting on coordinators’ time and capacity for other groups	• Working in areas already targeted for regeneration helped generate funding and facilitated some community projects that had been started but were making no or slow progress • Working with existing and established groups who are passionate about their local area • Having a specific issue to focus on identified by the community group • Having a key contact or champion in each community group facilitated communication and intervention delivery

Working in areas that the coordinator was not familiar with was thought to be a challenge: “…*the more difficult challenge was working in [town] where I don’t live” (FG, 2011).* Coordinators indicated that *“it really helps if you know the areas and you’ve got an idea of what the issues are likely to be and you can go out and speak knowledgably about the area” (Interview, 2011).* In addition, each LA area was different and functioned differently including their relationships with local communities:

*“Each local authority area is very different and the whole set up of the counties is very different. So there’s a lot of need to understand how they work and who’s actually engaged with local people, it’s good to get an understanding of how each council works.”(FG, 2008)*

The delivery of the intervention varied between LA areas depending on the internal structure of the LA, their understanding of and commitment to the intervention, the role of the main contact in the LA (and their capacity); working processes of the LA, communication channels across LA departments; the existence of a neighbourhood management team; and the funding available. Coordinators reported that the main contact in the LA was a key influence on what was delivered during the intervention regarding LA-led environmental improvements and that it is important to find the right person for this role. Factors to consider included the relevance of their role to the walking agenda, capacity to undertake activities required for the intervention, and ability/authority to act on the audit reports received and identify funding and work capacity to undertake improvements.

The flexibility for coordinators to adapt delivery of the intervention based on local context, partners, resources, funding and needs was an important aspect facilitating the implementation of the intervention.

*“We have got a common project but we have all put different spins on it and for me, that’s an important part because different things work in different areas.” (Coordinator, 2009)*

In a number of communities, the group who registered for FFW had already identified an area that they wanted to improve prior to the intervention but were struggling to make progress, or had already started making some improvements but progress was slow. The coordinators felt that FFW had built on this and they had facilitated the groups to start work or speeded up the processes needed to make the improvements. The groups which were recruited to FFW were generally already established and active in their communities which facilitated intervention implementation.

Although the delivery evolved during the intervention and became more standardised as the intervention progressed, some of the coordinators’ expectations at the start were not met due to the barriers which were encountered. In particular, coordinators’ capacity to work with multiple groups across multiple areas, their ability to undertake CSAs in all projects, the LA’s capacity to fund and implement recommendations, long timeframes for making LA-led environmental improvements, timescales for working with each community group, ability to exit from or close projects, and targets set by the funder (for the number of groups the coordinators should work with and the number of individuals which should be engaged in the intervention) all influenced, both positively and negatively, the implementation of the intervention. This included the number and types of community groups coordinators were able to work with (which was lower than expected), the introduction of ‘secondary groups’ (which helped to expand the reach of each community project and meet funder targets), the number of projects where CSAs were conducted and LA-led environmental improvements were implemented (which were much lower than expected), and the development of resources e.g. pledge cards, (which were introduced later to increase reach within the wider community to meet funder targets, but actually became a useful engagement tool).

*“I think when I first started, I tried to keep it as pure as I could and stick to the remit of the project as purely as I could by working with community groups and looking at ways to improve the walkability of their area. But over time, because the timeframes involved in making recommendations and putting reports in and work getting done, because some of the stuff I was doing last year is only just having an effect this year. So twelve, eighteen months on, it’s still in the process and not even been delivered yet. So with that in mind, I think that it has changed for me because every project can’t all be about doing audits, putting reports in, making recommendations, because there just isn’t time. So the projects have to be more diverse and they’ve had to look… not away from the remit of the project I think, but had to look more widely and encompass the groups where I know, even when I start with them, that it’s not going to involve an audit. And we’re not going to make recommendations but we just walked or we’ve just done the community clean-up and maybe not an audit, but I’ve tried to keep it pure and I thought this is what it’s about, it’s working with communities and it’s trying to deliver change on the ground and improvement to walking levels.” (FG, 2009)*

At the start of the intervention no promotional materials had been developed which proved challenging for publicising the intervention:

*“The first thing I did was get myself a stall there, we had very little paraphernalia, very little literature but we had little bits of stuff, so I was just kind of stood there giving my card out, really talking to people saying that, “You know, there’s this new project, it’s just about starting, getting started in [town],” but, you know, I didn’t have much to show anyone, I didn’t have any kind of case studies or anything really, I was just kind of telling them about what this project might be and how it might benefit people.” (Interview, 2011)*

Although the coordinators initially reported that the transport department was the most appropriate department to engage with in the LA, it became clear later that this was not always the best department through which to work with communities and that other departments or partners were needed in order to support this aspect of the intervention:

*“You can’t really reach neighbourhoods through the transport team because they don’t really have any community contacts but I used the neighbourhood managers or coordinators to access local issues and tenants’ and residents’ associations or other local groups, and then, you know, you just work with them on the ground to try and achieve those improvements and then flag up things to the transport team where they can support it.” (Interview, 2011)*

The timescales for engaging community groups, delivering the intervention and for LA-led environmental changes to be implemented was much longer than expected, and keeping groups engaged in the intervention during this time was a challenge:

*“It’s getting [audit recommendations] done in a timescale that people haven’t forgotten about it because it’s not realistic to get most of them done in 3 or 4 months or even 6 months because they’re just, that’s not how things work. But for communities 3 or 4 months is just absolutely ages and they’ve completely forgotten about the audits.” (Interview, 2010)*

There were some challenges with regards to funding intervention activities. Although the LA provided funding for small scale infrastructural improvements, some activities fell outside of the LA remit or were not on LA-owned land therefore additional funding had to be sourced from other local organisations. During FFW there was a period of significant economic downturn in the UK. LA budget cuts during the intervention impacted on funding available for FFW and once match funding had been achieved there was little further investment in the intervention in some LA areas. The economic downturn may also have affected local investment in FFW through reduced funding availability in other local organisations and partners. The coordinators also had a budget which could be used for activities which helped to engage LAs, local organisations and communities in the intervention and to support intervention delivery. In some areas it was possible to attract additional funding through being in a regeneration area or through other local organisations e.g. housing associations or neighbourhood management.

*“There’s quite a lot of long-term urban regeneration going on and there’s funding available for that. We’re hoping that by being hopefully in that area and quite high profile, that then can tie into their aims of the regeneration and steer funding.” (FG, 2008)*

Ending projects and involvement with a community group was one of the main challenges: “*I think probably a biggest challenge and maybe that’s because we don’t have a Fitter for Walking award yet, is closing off a community” (Interview, 2009)*. Coordinators became very closely involved with many communities*,* and it was difficult to end the relationship. The development of the FFW award was reported to be extremely helpful in addressing this issue as it helped to ‘finalise’ the input from the coordinators and close the project.

## Discussion

This paper presents an evaluation of the implementation of the FFW intervention, which was designed to change the environment at the street scale level to promote walking for transport in deprived areas with low levels of physical activity. The study was based on the principles of implementation research [29, 37] and key constructs were assessed including reach (recruitment and participation), dosage (what was delivered), implementation processes and adaptation (how the intervention was delivered) and factors influencing implementation (barriers and facilitators). The findings show that this was a complex intervention to implement and there were a number of factors which influenced reach, dosage, implementation processes and adaptation.

In terms of reach, 155 community groups engaged with FFW thus the target of recruiting 228 community groups was not met. Evidence from this study suggests this can be explained by a number of barriers which were identified in the implementation processes. Coordinator capacity to work with multiple groups and undertake full CSAs, LA resources and funding, and the timescales taken to make environmental improvements (which meant coordinators stayed involved with groups for much longer than expected) were the main limiting factors. These factors need to be taken into consideration in planning future interventions of this type, in particular setting realistic targets, ensuring there is sufficient coordinator capacity to work with multiple groups, and securing resources and funding from the LA. The characteristics of the groups and those of group members were not recorded, however the coordinators noted that the groups which did sign up were generally already well established and some were already attempting to address issues in their local neighbourhood environment. Working in areas where there are no existing community groups, or with groups for whom the neighbourhood environment and walking is not a priority, may therefore be much more challenging.

The results show that a large number of individuals engaged with FFW, and many were encouraged to walk more during the intervention, suggesting it was a popular intervention with local communities. Interventions which reach large numbers of individuals are thought to be important for public health impact even if the effects of the intervention are only small [38, 39]. Thus FFW shows promise as an intervention with a large reach, particularly in deprived communities where ‘hard-to-reach’ groups exist. However, the demographic characteristics and physical activity levels of these participants were not recorded, therefore it was not possible to assess the representativeness of participants or determine whether the target group (those with low levels of physical activity) were reached. It is possible that those who engaged were already interested in being active or walking, or were involved in existing community activities.

FFW aimed to improve the local neighbourhood environment at the street scale level. The results show that it was possible to implement these types of environmental changes. The most frequently reported LA-led changes were related to removing encroaching vegetation, installing new signage, new or improved dropped kerb crossings and new or improved footpaths. Community-led changes mainly focussed on improving aesthetics such as planting bulbs or cleaning up streets or local areas. These types of changes are similar to street scale changes made in other interventions [21, 24] and appear to be feasible to deliver when using a community engagement approach, potentially eliminating local barriers to walking for transport. However, in terms of dosage, there was wide variation in what was delivered in each project in relation to environmental changes. The results also show that LA-led changes were not undertaken in all projects, and in 32 of the projects no environmental improvements were undertaken at all. Evidence from discussions with the coordinators suggests this can be explained by a number of factors including the needs, interests and skills of the community group, coordinator capacity, LA resources and funding, the timescales to make environmental improvements (some projects had planned environmental improvements but they had not been implemented at the time of final data collection), and the addition of secondary groups which were introduced to extend the reach of the project and promote the newly improved routes, rather than undertake environmental improvements. These factors need to be taken into consideration in planning future interventions.

In many projects there was a focus on using promotional and awareness-raising activities to complement environmental improvements, or in some cases instead of environmental improvements. These activities were reported to play an important role in the intervention for initially engaging community groups, maintaining momentum whilst waiting for environmental changes to be made, increasing awareness of walking and local routes, promoting environmental improvements, and engaging wider members of the community to extend the reach of the intervention. Thus others developing similar interventions may wish to consider using these types of approaches to engage communities or to complement other intervention activities. The need for promotional and awareness-raising activities to complement environmental improvements in order to effectively increase walking levels, compared to making environmental improvements alone, is not yet fully understood and requires further investigation [40].

In FFW, there was no prescribed implementation strategy at the start of the intervention making it difficult to make any assessment of whether the intervention was delivered as intended. There was however an overarching plan to deliver some key elements. Processes were identified for the implementation of the FFW intervention, including the need for a preparatory phase and a delivery phase involving a four step process, with flexibility to adapt implementation at the local level. The importance of adaptability for implementing an intervention, defined as “the degree to which an intervention can be adapted, tailored, refined, or reinvented to meet local needs”, has previously been highlighted [41, 42]. The findings from this study demonstrate that adaptability was critical due to differences in local contexts, the way in which the LAs operated, the needs and interests of the community groups engaged and differences in local neighbourhood environments. Future interventions of this type should allow for adaptability at the local level, whilst maintaining core principles in the overall implementation strategy.

The development of partnerships has been identified as a good practice characteristic for interventions aiming to change physical activity behaviour in order to facilitate adoption and implementation, and has also been acknowledged as essential for planning and promotional efforts to increase physical activity [22, 43]. FFW relied heavily on partnerships with the LA, other local organisations and community groups to implement the intervention. However, the findings show there were a number of barriers in doing so which need to be addressed in future. In particular, the time needed to develop partnerships before commencing intervention delivery was identified, which has also been reported elsewhere when using this type of approach to deliver community-based interventions to promote walking [23].

One of the key implementation strategies in FFW was engaging with community groups and consulting with them to identify barriers to walking in their local area, along with proposing solutions. The findings showed that this was a challenging and time consuming process, however it led to the implementation of LA-led and community-led environmental improvements in some of the projects. The importance of gaining community buy-in, and in particular involving communities in deprived areas in developing intervention content, has been highlighted elsewhere [22, 44]. In addition, previous research has shown the benefits of using an asset-based approach, such as that applied in FFW, whereby local communities identify barriers and solutions to health issues themselves [23]. This approach therefore warrants use in future interventions, whilst taking note of the capacity and resource needed for implementation.

A large number of barriers and facilitators were identified in relation to the implementation of FFW. This study adds to a modest body of existing knowledge regarding the factors influencing the implementation of interventions which promote walking. The National Institute for Health and Care Excellence reported evidence from four studies regarding barriers and facilitators for planning and delivering community-based walking interventions [23]. However, these studies did not include environmental improvements and most did not undertake a full evaluation of the implementation of the intervention. The main facilitators identified were organisational support (including provision of promotional material), collaboration with local partners and the importance of partners understanding goals. In contrast, barriers included the short amount of time in which to establish partnerships before commencing intervention delivery, time taken for recruitment, lack of inter-organisational collaboration and not having clarity about the intervention goals. The role of a coordinator in facilitating relationships between partners was also highlighted. Similar findings were observed in the present study confirming that these factors should be considered in the planning and delivery of future interventions.

The need for interventions which improve the built environment to support and promote walking for transport has been highlighted [24]. FFW is one of few reported interventions which have aimed to use a community engagement approach to change the environment at the street scale level to promote walking for transport, or to comprehensively evaluate the implementation of this type of approach, addressing a gap in the literature. In addition, FFW targeted deprived areas which often have neglected local environments which de-motivate people from walking [15]. A number of barriers to walking were identified by community groups taking part in FFW, and environmental improvements successfully implemented, thus addressing the poor environment and providing safe, attractive and enjoyable settings for walking in these areas. The learning from this study helps to identify how these types of intervention can be delivered in deprived areas and barriers which may need to be overcome for effective implementation.

FFW was a practice-led intervention; researchers were not involved in intervention planning and no specific intervention model or behaviour change theory was used to inform intervention development. A recent review of physical activity interventions in socio-economically disadvantaged communities found that interventions which were underpinned by a theoretical framework were more likely to be effective compared to those which were not [38]. In future, developing these types of real world intervention may benefit from using co-production approaches which involve researchers, as well as those developing and implementing the intervention, community representatives and stakeholders, to facilitate an evidence-based and theory-driven approach to the intervention design. Such theory might include the socio-ecological model to inform the development of multi-level interventions [14, 45], or specific behaviour change techniques known to influence walking [46].

Implementation research in the field of physical activity and public health is in its infancy. This study develops the evidence base regarding the implementation processes required, and the barriers which need to be overcome, in effectively delivering this type of intervention in a real world setting. The findings from this study will facilitate future research into these types of interventions along with supporting replication of the intervention, scale-up and embedment into local systems. In addition, the UK Government recently launched its’ first Cycling and Walking investment strategy which aims to make walking (and cycling) the norm for shorter journeys, or as part of longer journeys by 2040 [47]. As part of this strategy there is an ambition to improve safety and connectivity, reduce traffic speeds, develop more ‘walkable’ areas, install safe paths and improve public realm. The FFW intervention approach may offer a potential (part) solution for delivering this ambition and in making changes that are needed to improve local street environments, particularly in deprived areas. However it should be noted that undertaking community engagement and improving street environments can be complex, and resource and time intensive, therefore substantial funding may be required to undertake this type of intervention. Although FFW targeted deprived areas, it is likely the principles of implementation described in this study are generalisable and could be transferred to other geographical areas. However, adaptation to local context, specific setting and target population group remains a key factor in successful implementation and this has been highlighted previously [22].

### Recommendations

**Fig. 2:**
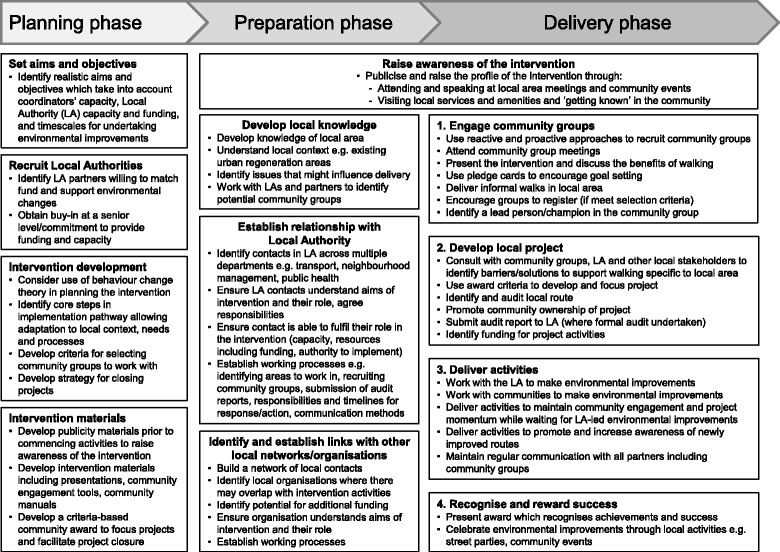
Summary of implementation processes for improving street environments to promote walking for transport

Understanding what was delivered, and how, in an intervention can “provide policy makers and practitioners with vital information about how the intervention might be replicated, as well as generalisable knowledge on how to implement complex interventions” [48]. Based on the findings from this study, a set of implementation recommendations (outlined below) and a summary framework of the processes required for implementation (Fig. 2) are proposed which could be used to replicate and scale-up the intervention. The figure includes the processes required during an additional planning phase which is suggested in order to facilitate implementation of the preparation and delivery phases.

These recommendations will help researchers, practitioners and policy makers (including LAs and other organisations interested in promoting walking) to identify the key steps which are required to implement interventions which use a community engagement approach to change the environment to promote walking for transport in deprived areas. In addition, the recommendations should help with making realistic plans for what can be achieved when using this type of approach, and the resource, capacity and timescales which might be needed for successful implementation. The recommendations, principles and processes may also be applied to other community-based physical activity interventions which use community engagement and partnership approaches.

#### For intervention developers (research and practice)

1. The structure of the intervention should have core components relating to implementation which allow flexibility in how they are delivered so they can be adapted and tailored to suit local context, processes and needs.
2. Promotional materials for use in publicising and increasing awareness of the intervention (and walking) should be developed prior to starting these activities to facilitate engagement of local organisations and community groups.
3. In planning programmes such as these, realistic targets should be set regarding the size and number of areas, and the number of communities, it will be possible to work with, taking into account the staff resource needed to undertake community engagement activities and the time needed to understand the local context.
4. Criteria should be developed for selecting communities to work with, as well as setting criteria for the communities to work towards, to ensure that appropriate community groups are recruited who have a clear understanding of the purpose and remit of the project. This will avoid the project being used to address other agendas or being seen as an opportunity purely for led walks.
5. Communities should be consulted to ensure the environmental improvements made and activities delivered meet local needs and interests. Community members should be engaged in identifying the barriers to walking to ensure the intervention activities are community-led and to facilitate the community taking ownership for their local walking environment. Community Street Audits could be used to undertake this consultation however sufficient resource needs to be provided in terms of coordinator and LA capacity to undertake the audits and respond to the findings and recommendations.
6. An award should be developed which requires specific criteria to be met to help focus the community on delivering specific objectives in their project, to celebrate community achievements and recognise success, and to facilitate a natural end to project support.
7. Funding may need to be sourced from outside of the LA to make environmental improvements where budgets are limited, or where the LA does not own the land where improvements are required. Coordinators should make links with local regeneration programmes or with other local organisations to facilitate access to this funding.

#### For coordinators (implementers)

8. A minimum 6 month preparatory phase is needed to familiarise with the local area; identify contextual factors that might influence implementation; understand local issues (including identifying areas which are already part of local regeneration programmes); and establish partnerships and working practices. Coordinators should attend local area meetings and community events to build contacts early on to raise the profile of themselves and the intervention.
9. The coordinator should familiarise themselves with the LA and understand how it operates (including communication channels between departments); identify relevant departments in the LA which may have a role in supporting the intervention, either in selecting and meeting with communities/community groups, or funding and implementing environmental improvements (e.g. transport, neighbourhood management, environment, housing, regeneration and economic development or public health) and develop a network of contacts; ensure the LA understands the intervention and their role; establish responsibilities and processes for the intervention with the LA including identification of communities and submission of CSA reports. Ensure the primary contact is able to act on any CSA reports which are submitted, or can disseminate to the correct contact/department in the LA.
10. Coordinators should identify other potential relevant local organisations, develop relationships and build a network of local contacts that can support implementation of the intervention (and potentially provide access to additional funding).
11. Coordinators should maintain communication and keep all stakeholders up to date with progress on a regular basis including LAs, other partner organisations and community groups.

#### For those working with communities

12. It should be made clear at the outset that the community group is expected to take leadership and ownership of activities to reduce the burden on the coordinator and promote long-term sustainability.
13. A key contact or champion should be identified within each community group to facilitate communication and implementation.
14. Community expectations should be managed to ensure they understand processes and timescales for undertaking LA-led environmental improvements and are provided with a realistic sense of what might be implemented and when.
15. Whilst waiting for improvements to be made, additional activities may need to be implemented to maintain momentum, and community engagement and involvement in the project. Examples might include community-led environmental improvements e.g. litter picks or clean up days and other promotional or awareness-raising activities e.g. led walks.
16. Community-led environmental changes should be promoted such as litter picks and planting bulbs as these can play an important role in improving the local environment to promote walking, and in bringing the community together, particularly where limited funding is available.

#### For local authorities

17. Ensure there is: senior management support for the intervention; clarity and understanding regarding the aims and objectives of the intervention; clearly defined roles and responsibilities; and an understanding of expectations from other partners.
18. Establish partnerships across departments within the LA, e.g. transport, neighbourhood management, environment, housing, regeneration and economic development and public health, to facilitate intervention implementation and communication regarding intervention activities.
19. Identify a primary contact whose role fits with remit of the intervention and who has the authority to be able to deliver the LA component of the intervention. This should include making decisions based on audit recommendations and arranging for environmental improvements to be undertaken.
20. Ensure there are processes in place to respond to audit recommendations, there is sufficient funding available to undertake the environmental improvements requested, and workforce capacity to undertake the environmental improvements.

### Strengths and limitations

Undertaking interviews and focus groups with the coordinators at regular intervals during FFW is a strength of the research and helped to assess the development of the intervention as it evolved, as well as to identify phases and processes for implementation of the intervention. The views of the LAs and participants have not been taken into consideration in this study, which may present some bias in the findings. The implementation logs provided a useful resource as a real time evaluation tool for characterising and reporting on the implementation of the intervention. Although most coordinators completed the implementation logs in great detail, one or two coordinators provided less information and some details may have been missing which is a limitation of this study. It was not possible to evaluate quantitatively the level of intervention delivered in each area with the methods used in this evaluation. Due to budgetary constraints the characteristics of community groups and individuals who participated in FFW (and their representativeness) were not recorded. In addition, the impact of the intervention on individual behaviour change with regards to walking and physical activity was not assessed (also due to budgetary constraints for the evaluation), other than route user counts and surveys in five projects [35]. Therefore it was not possible to determine the overall effectiveness of different levels of the intervention on individual walking levels in each of the different projects and this warrants further investigation.

## Conclusion

FFW is one of few interventions which have used a community engagement approach to change the street environment to promote walking for transport, or which have targeted deprived areas with this strategy. There has also been limited evaluation to date of the implementation processes required when using this type of approach. A substantial number of community groups and individuals engaged in FFW indicating the intervention has potential for a large reach and thus population impact. A range of street scale environment improvements were made by LAs and communities alongside promotional and awareness-raising activities. However, environmental changes were not undertaken in all projects due to a number of barriers to implementation. Delivering LA-led improvements requires coordinator and LA capacity and funding, and can take some time to implement, reducing the number of communities it may be possible to work with. Promotional and awareness-raising activities have an important role in engaging communities, promoting newly improved routes and extending the reach of intervention activities. Implementing this type of intervention is complex and a number of barriers and facilitators need to be overcome for optimal delivery. The findings from this study address a gap in the literature regarding the understanding of the implementation of these types of physical activity promotion strategies. They helped to inform the development of a set of recommendations and a summary of implementation processes for future interventions using a community engagement to improve the street environment to promote walking for transport.

## Additional files

Additional file 1: Physical activity levels and indices of multiple deprivation (IMD) for participating local authorities. (DOCX 18 kb)

Additional file 2: Types of activities delivered in Fitter for Walking. (DOCX 19 kb)

## Abbreviations

CSA: Community Street Audit
FFW: Fitter for Walking
LA: Local authority
