# Supplementary material for: Evaluation of the implementation of an intervention to improve the street environment and promote walking for transport in deprived neighbourhoods
Source: BMC Public Health. 2017 Aug 14;17:655. doi: 10.1186/s12889-017-4637-5 (PMC5557560; doi:10.1186/s12889-017-4637-5)
Supplement: Supplementary file 1 — Physical activity levels and indices of multiple deprivation (IMD) for participating local authorities. (DOCX 18 kb) [file 12889_2017_4637_MOESM1_ESM.docx]

**Additional File 1. Physical activity levels and indices of multiple deprivation (IMD) for participating local authorities**

**Table A1. Physical activity levels and IMD scores in local authorities recruited for Fitter for Walking**

| **Region** | **Local Authority Area** | **% participating in physical activity^ab^** | **Summary IMD score across LA^c^** | **Rank based on average score^d^** |
| --- | --- | --- | --- | --- |
| London | Barking & Dagenham | 14.6 | 34.5 | 22 |
|  | Redbridge | 15.1 | 20.4 | 143 |
| North East England | Gateshead | 16.2 | 29.5 | 52 |
|  | Newcastle | 22.1 | 31.4 | 37 |
|  | Sunderland | 20.7 | 31.8 | 35 |
| North West England | Blackburn with Darwen | 14.9 | 35.8 | 17 |
|  | Bolton | 15.2 | 29.7 | 51 |
| West Midlands | Dudley | 15.1 | 23.7 | 100 |
|  | Sandwell | 14.8 | 37.0 | 14 |
|  | Wolverhampton | 13.2 | 33.0 | 28 |
| Yorkshire | Doncaster | 14.5 | 30.8 | 41 |
|  | Rotherham | 19.9 | 26.7 | 68 |

^a^ Data source: Active People Survey (Oct 2005-April 2006)

^b^ Participating in physical activity is defined as participation in at least 30 minutes moderate intensity sport and active recreation (including recreational walking) on 3 or more days a week

^c^Summary IMD (index of multiple deprivation) score for LA (local authority) is the average across the Lower Super Output Area level for that LA area.

^d^The rank is based on the summary IMD score: 1=highest area of deprivation; 354 =least deprived

(data source: <http://webarchive.nationalarchives.gov.uk/+/http://communities.gov.uk/communities/neighbourhoodrenewal/deprivation/deprivation07/>)
