# Supplementary material for: Evaluation of the implementation of an intervention to improve the street environment and promote walking for transport in deprived neighbourhoods
Source: BMC Public Health. 2017 Aug 14;17:655. doi: 10.1186/s12889-017-4637-5 (PMC5557560; doi:10.1186/s12889-017-4637-5)
Supplement: Supplementary file 2 — Types of activities delivered in Fitter for Walking. (DOCX 19 kb) [file 12889_2017_4637_MOESM2_ESM.docx]

**Additional File 2. Types of activities delivered in Fitter for Walking**

**Table A2.1 Local authority-led environmental changes**

| **Theme** | **Activity** | **Number of projects** |
| --- | --- | --- |
| Signage | New or improved pedestrian signage | 9 |
|  | Installation of map/notice board for map | 5 |
| Clearance / improved access | Clearing vegetation /encroaching vegetation / overgrowth | 12 |
|  | Site clearance and general improvement | 2 |
| Street furniture | Removal/repair/replacement of street clutter/furniture | 5 |
| Improved crossings | Dropped kerbs / kerb improvements | 8 |
|  | New/improved crossings | 4 |
| Traffic management | Signage for traffic to reduce speed | 2 |
|  | 20 mph zone / signs | 3 |
|  | Extra bollards for traffic management and prevention e.g. parking control | 6 |
|  | Traffic calming | 2 |
|  | Other parking improvements (reduction in pavement parking) | 1 |
| Footpath/pavement improvements | New/repaired/improved footpaths/pavements | 8 |
|  | Resurfacing of footpaths/pavements | 8 |
|  | Widened footpaths/pavements | 2 |
|  | Extensions to footpaths/pavements | 2 |
| Safety | Street lighting (new or improved) | 5 |
|  | General safety improvements e.g. new guard railing; new fencing; repairs to bridge; remove dangerous steps | 8 |
| Litter and graffiti | Installation of new litter bin | 3 |
|  | Installation/relocation of dog bins | 2 |
|  | Removal of graffiti | 3 |
| Seating | Seating/bench installed | 6 |
| General aesthetics | Gateway improvements on to route | 3 |
|  | New bus shelter | 1 |
|  | Planters removed and replaced | 1 |
|  | Tidying of gardens/allotments | 1 |
| Other LA actions | Arranged clean-up day | 1 |
|  | Raised capacity for action (e.g. royal navy to support clean up) | 1 |
|  | Use of community payback | 3 |
|  | Council enforcement and traffic road orders (e.g. to control parking) | 3 |
|  | Investigate drainage issues | 1 |

**Table A2.2 Community/co-ordinator-led environmental changes**

| **Theme** | **Activity** | **Number of projects** |
| --- | --- | --- |
| Cleaning and tidying | Clean up days | 12 |
|  | Litter pick up | 8 |
|  | Clearance of untidy ground/path clearance/vegetation | 7 |
|  | Removal of graffiti | 2 |
|  | Removal of waste from local residents gardens | 2 |
| Improvements | Planting of bulbs/bedding plants/shrubs/wild flowers | 33 |
|  | Garden projects (e.g. school, church grounds) | 2 |

**Table A3.3 Promotional and awareness-raising activities**

| **Theme** | **Activity** | **Number of projects** |
| --- | --- | --- |
| Information and resources | Map development / information leaflets (art projects) / provision of route information | 22 |
| Walks | Led walks | 60 |
|  | Themed walks (e.g. nature walks, treasure hunts, lantern walks, bat walks, bird walks) | 18 |
|  | School walking trips / walkabout events (with children) | 5 |
| Pledge cards | Pledge cards (often used in schools with children/parents and tied into walking challenges) | 17 |
| Community events | Community events and fun days (stalls with resources, activities and pledge cards) | 12 |
|  | Celebrating improvements (official opening of improvements / launch event / street parties) | 7 |
|  | Art workshops/craft activities /project with children re road safety and walking themed / poster competition | 6 |
| School-based activities | Talks/assemblies with school children (and/or parents) – walking and safety | 19 |
|  | Walking challenges (schools/families) and walking activities linked to campaigns e.g. walk to school month / walk to school week / pedometer challenge | 12 |
|  | Activities at school (classroom or after school) | 8 |
|  | Activities with parents | 1 |
